# 2-Chloro-4-(2-iodo­benzene­sulfonamido)­benzoic acid

**DOI:** 10.1107/S1600536811016412

**Published:** 2011-05-07

**Authors:** Muhammad Nadeem Arshad, Islam Ullah Khan, H. M. Rafique, Abdullah M. Asiri, Muhammad Shafiq

**Affiliations:** a X-ray Diffraction and Crystallography Laboratory, Department of Physics, School of Physical Sciences, University of the Punjab, Quaid-e-Azam Campus, Lahore 54590, Pakistan; b Materials Chemistry Laboratory, Department of Chemistry, GC University, Lahore 54000, Pakistan; c The Center of Excellence for Advanced Materials Research, King Abdul Aziz University, Jeddah, PO Box 80203, Saudi Arabia

## Abstract

In the title compound, C_13_H_9_ClINO_4_S, the dihedral angle between the aromatic rings is 81.04 (17)°. The disposition of the I and Cl atoms attached to the two rings is *anti*. In the crystal, mol­ecules are connected *via* O—H⋯O and N—H⋯O hydrogen bonds.

## Related literature

For background to thia­zine heterocycles, see: Arshad *et al.* (2008, ▶ 2011 ▶). For their biological activity, see: Medina *et al.* (1999 ▶). For related structures, see: Arshad *et al.* (2009*a* ▶,*b* ▶,*c* ▶).
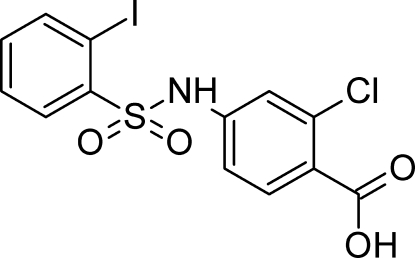


## Experimental

### 

#### Crystal data

C_13_H_9_ClINO_4_S
*M* *_r_* = 437.62
Monoclinic, 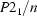

*a* = 14.1522 (8) Å
*b* = 7.3203 (4) Å
*c* = 14.7193 (8) Å
β = 104.892 (2)°
*V* = 1473.68 (14) Å^3^
*Z* = 4
Mo *K*α radiation
μ = 2.51 mm^−1^
*T* = 296 K
0.18 × 0.15 × 0.09 mm

#### Data collection

Bruker Kappa APEXII CCD diffractometer
Absorption correction: multi-scan (*SADABS*; Bruker, 2007 ▶) *T*_min_ = 0.661, *T*_max_ = 0.806
16645 measured reflections
3668 independent reflections
1876 reflections with *I* > 2σ(*I*)
*R*_int_ = 0.058

#### Refinement

*R*[*F* ^2^ > 2σ(*F* ^2^)] = 0.054
*wR*(*F* ^2^) = 0.129
*S* = 1.03
3668 reflections
191 parameters
H-atom parameters constrained
Δρ_max_ = 1.24 e Å^−3^
Δρ_min_ = −1.30 e Å^−3^

### 

Data collection: *APEX2* (Bruker, 2007 ▶); cell refinement: *SAINT* (Bruker, 2007 ▶); data reduction: *SAINT*; program(s) used to solve structure: *SHELXS97* (Sheldrick, 2008 ▶); program(s) used to refine structure: *SHELXL97* (Sheldrick, 2008 ▶); molecular graphics: *ORTEP-3* (Farrugia, 1997 ▶) and *PLATON* (Spek, 2009 ▶); software used to prepare material for publication: *WinGX* (Farrugia, 1999 ▶) and *PLATON*.

## Supplementary Material

Crystal structure: contains datablocks I, global. DOI: 10.1107/S1600536811016412/hb5865sup1.cif

Structure factors: contains datablocks I. DOI: 10.1107/S1600536811016412/hb5865Isup2.hkl

Supplementary material file. DOI: 10.1107/S1600536811016412/hb5865Isup3.cml

Additional supplementary materials:  crystallographic information; 3D view; checkCIF report

## Figures and Tables

**Table 1:** Hydrogen-bond geometry (Å, °)

*D*—H⋯*A*	*D*—H	H⋯*A*	*D*⋯*A*	*D*—H⋯*A*
N1—H1⋯O2^i^	0.86	2.38	3.214 (6)	162
O1—H1*O*⋯O2^ii^	0.82	2.09	2.771 (6)	140

Symmetry codes: (i) 
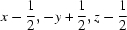
; (ii) 
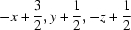
.

**Fig. 1.**
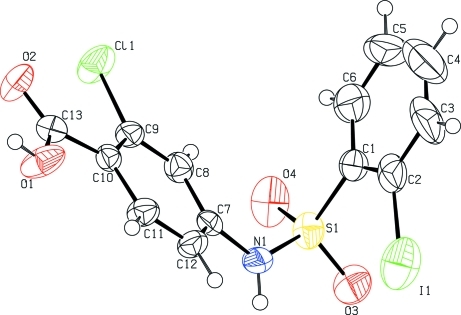
The structure of (I) with displacement ellipsoids drawn at the 50% probability level.

**Fig. 2.**
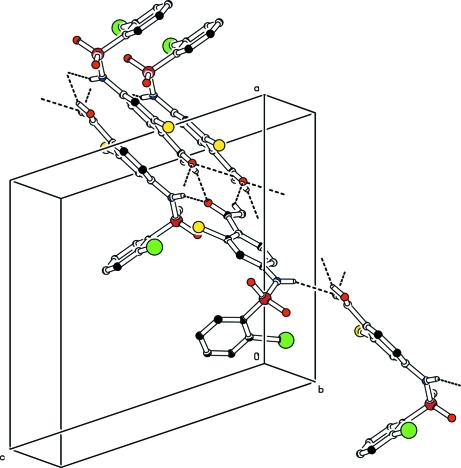
Unit cell packing for (I) with hydrogen bonding shown as dashed lines.

## supplementary crystallographic information

### Comment

The title compound, (I), is an example of a halogenated sulfonamide, which are reported as inhibitors for the growth of multidrug resistant MCF-7/ADR cancer cells (Medina *et al.*, 1999). In continuation to our researches with sulfonamides (Arshad *et al.*, 2009*a*), the title compound has been prepared, as an intermediate for the preparation of thiazine related heterocycles (Arshad *et al.*, 2008, 2011).

The crystal structure of (I) being reported here as an isomer of previously reported compound 2-chloro-5-(2-iodobenzenesulfonamido)benzoic acid (II), (Arshad, *et al.*, 2009*a*). The bond lengths and bond angles are also compareable with the 5-benzenesulfonamido-2-chlorobenzoic acid (III), (Arshad, *et al.*, 2009*b*) and 4-(2-Iodobenzenesulfonamido)benzoic acid monohydrate (IV) (Arshad, *et al.*, 2009*c*). The two aromatic rings A (C1-C6) and B (C7-C12) are oriented at dihedral angle of 81.04 (17)° which is lower than in (IV i.e. 87.07 (6)°) and greater than in (II i.e. 74.46 (9)°) and (III i.e. 74.18 (17)°). No intramolecular hydrogen bonding interaction have been observed in the molecule. The intermolecular interaction of O—H···O and N—H···O forms three dimentional network to stabilize structure of molecule (Fig. 2 and Tab. 1).

### Experimental

The title compound was prepared following the method of Arshad *et al.* (2009*a*). Red needles were recrystallised from methanol.

### Refinement

All the H-atoms were positioned with idealized geometry with C—H = 0.93 Å, N—H = 0.86 Å, O—H = 0.82 Å and were refined using a riding model with *U*_iso_(H) = 1.2 *U*_eq_for aromatic C and N atoms and with *U*_iso_(H) = 1.5 *U*_eq_for O atom.

### Crystal data

**Table:**

C_13_H_9_ClINO_4_S	*F*(000) = 848
*M**_r_* = 437.62	*D*_x_ = 1.972 Mg m^−^^3^
Monoclinic, *P*2_1_/*n*	Mo *K*α radiation, λ = 0.71073 Å
Hall symbol: -P 2yn	Cell parameters from 2692 reflections
*a* = 14.1522 (8) Å	θ = 2.9–22.2°
*b* = 7.3203 (4) Å	µ = 2.51 mm^−^^1^
*c* = 14.7193 (8) Å	*T* = 296 K
β = 104.892 (2)°	Needle, red
*V* = 1473.68 (14) Å^3^	0.18 × 0.15 × 0.09 mm
*Z* = 4	

### Data collection

**Table:**

Bruker Kappa APEXII CCD diffractometer	3668 independent reflections
Radiation source: fine-focus sealed tube	1876 reflections with *I* > 2σ(*I*)
graphite	*R*_int_ = 0.058
φ and ω scans	θ_max_ = 28.3°, θ_min_ = 2.9°
Absorption correction: multi-scan (*SADABS*; Bruker, 2007)	*h* = −18→18
*T*_min_ = 0.661, *T*_max_ = 0.806	*k* = −9→5
16645 measured reflections	*l* = −19→18

### Refinement

**Table:**

Refinement on *F*^2^	Primary atom site location: structure-invariant direct methods
Least-squares matrix: full	Secondary atom site location: difference Fourier map
*R*[*F*^2^ > 2σ(*F*^2^)] = 0.054	Hydrogen site location: inferred from neighbouring sites
*wR*(*F*^2^) = 0.129	H-atom parameters constrained
*S* = 1.03	*w* = 1/[σ^2^(*F*_o_^2^) + (0.0383*P*)^2^ + 3.6338*P*] where *P* = (*F*_o_^2^ + 2*F*_c_^2^)/3
3668 reflections	(Δ/σ)_max_ < 0.001
191 parameters	Δρ_max_ = 1.24 e Å^−^^3^
0 restraints	Δρ_min_ = −1.30 e Å^−^^3^

### Special details

**Table:**

Geometry. All s.u.'s (except the s.u. in the dihedral angle between two l.s. planes) are estimated using the full covariance matrix. The cell s.u.'s are taken into account individually in the estimation of s.u.'s in distances, angles and torsion angles; correlations between s.u.'s in cell parameters are only used when they are defined by crystal symmetry. An approximate (isotropic) treatment of cell s.u.'s is used for estimating s.u.'s involving l.s. planes.
Refinement. Refinement of F^2^ against ALL reflections. The weighted R-factor wR and goodness of fit S are based on F^2^, conventional R-factors R are based on F, with F set to zero for negative F^2^. The threshold expression of F^2^ > 2σ(F^2^) is used only for calculating R-factors(gt) etc. and is not relevant to the choice of reflections for refinement. R-factors based on F^2^ are statistically about twice as large as those based on F, and R- factors based on ALL data will be even larger.

### Fractional atomic coordinates and isotropic or equivalent isotropic displacement parameters (Å^2^)

**Table:**

	*x*	*y*	*z*	*U*_iso_*/*U*_eq_	
I1	0.09820 (4)	0.29186 (7)	−0.02615 (4)	0.0813 (2)	
Cl1	0.57812 (11)	−0.0907 (2)	0.24047 (12)	0.0606 (5)	
S1	0.22376 (11)	−0.1302 (2)	−0.02876 (11)	0.0446 (4)	
O2	0.6990 (3)	0.2380 (5)	0.2686 (3)	0.0522 (11)	
O3	0.1453 (3)	−0.1280 (6)	−0.1113 (3)	0.0608 (12)	
O4	0.2813 (3)	−0.2894 (6)	−0.0053 (4)	0.0685 (13)	
O1	0.6486 (3)	0.4890 (5)	0.1868 (3)	0.0587 (12)	
H1O	0.6958	0.5337	0.2247	0.088*	
N1	0.2946 (3)	0.0364 (6)	−0.0429 (3)	0.0415 (12)	
H1	0.2808	0.0897	−0.0967	0.050*	
C1	0.1801 (4)	−0.0725 (8)	0.0698 (4)	0.0443 (14)	
C2	0.1285 (4)	0.0854 (10)	0.0765 (5)	0.0549 (17)	
C3	0.0945 (5)	0.1169 (14)	0.1543 (6)	0.082 (3)	
H3	0.0604	0.2237	0.1588	0.099*	
C4	0.1104 (8)	−0.005 (2)	0.2231 (8)	0.116 (4)	
H4	0.0860	0.0174	0.2749	0.140*	
C5	0.1625 (7)	−0.1670 (17)	0.2205 (6)	0.102 (4)	
H5	0.1740	−0.2503	0.2698	0.122*	
C6	0.1957 (5)	−0.1974 (11)	0.1423 (6)	0.070 (2)	
H6	0.2295	−0.3047	0.1380	0.085*	
C7	0.3765 (4)	0.1009 (7)	0.0255 (4)	0.0315 (12)	
C8	0.4328 (4)	−0.0092 (7)	0.0936 (4)	0.0372 (13)	
H8	0.4155	−0.1309	0.0976	0.045*	
C9	0.5151 (4)	0.0594 (7)	0.1565 (4)	0.0350 (13)	
C10	0.5457 (4)	0.2393 (6)	0.1501 (4)	0.0297 (11)	
C11	0.4845 (4)	0.3480 (7)	0.0827 (4)	0.0390 (13)	
H11	0.5006	0.4704	0.0788	0.047*	
C12	0.4019 (4)	0.2832 (7)	0.0222 (4)	0.0384 (13)	
H12	0.3625	0.3611	−0.0214	0.046*	
C13	0.6385 (4)	0.3173 (7)	0.2090 (4)	0.0364 (13)	

### Atomic displacement parameters (Å^2^)

**Table:**

	*U*^11^	*U*^22^	*U*^33^	*U*^12^	*U*^13^	*U*^23^
I1	0.0650 (3)	0.0593 (3)	0.1146 (5)	0.0148 (2)	0.0143 (3)	−0.0042 (3)
Cl1	0.0512 (9)	0.0458 (8)	0.0741 (12)	0.0036 (7)	−0.0037 (8)	0.0291 (8)
S1	0.0403 (8)	0.0417 (8)	0.0510 (10)	−0.0072 (6)	0.0103 (8)	−0.0130 (7)
O2	0.038 (2)	0.048 (2)	0.059 (3)	0.0023 (18)	−0.010 (2)	0.007 (2)
O3	0.055 (3)	0.071 (3)	0.051 (3)	−0.018 (2)	0.003 (2)	−0.023 (2)
O4	0.060 (3)	0.039 (2)	0.105 (4)	−0.004 (2)	0.020 (3)	−0.013 (2)
O1	0.069 (3)	0.044 (2)	0.045 (3)	−0.019 (2)	−0.017 (2)	0.006 (2)
N1	0.037 (3)	0.058 (3)	0.027 (3)	−0.008 (2)	0.005 (2)	0.001 (2)
C1	0.034 (3)	0.057 (4)	0.041 (4)	−0.013 (3)	0.006 (3)	0.001 (3)
C2	0.034 (3)	0.077 (5)	0.053 (4)	−0.020 (3)	0.010 (3)	−0.020 (4)
C3	0.062 (5)	0.125 (7)	0.075 (6)	−0.020 (5)	0.045 (5)	−0.039 (6)
C4	0.091 (8)	0.201 (13)	0.070 (7)	−0.062 (8)	0.045 (6)	−0.020 (8)
C5	0.082 (7)	0.160 (10)	0.057 (6)	−0.047 (7)	0.009 (5)	0.040 (7)
C6	0.053 (4)	0.085 (5)	0.070 (5)	−0.016 (4)	0.009 (4)	0.016 (4)
C7	0.030 (3)	0.041 (3)	0.026 (3)	−0.002 (2)	0.013 (2)	−0.001 (2)
C8	0.037 (3)	0.035 (3)	0.042 (4)	0.000 (2)	0.013 (3)	0.003 (3)
C9	0.036 (3)	0.036 (3)	0.034 (3)	0.010 (2)	0.012 (3)	0.011 (2)
C10	0.029 (3)	0.031 (3)	0.031 (3)	0.002 (2)	0.011 (2)	0.000 (2)
C11	0.042 (3)	0.037 (3)	0.036 (3)	−0.002 (2)	0.006 (3)	0.005 (2)
C12	0.036 (3)	0.040 (3)	0.036 (3)	0.000 (2)	0.002 (3)	0.012 (3)
C13	0.041 (3)	0.038 (3)	0.031 (3)	−0.001 (2)	0.012 (3)	0.001 (2)

### Geometric parameters (Å, °)

**Table:**

I1—C2	2.102 (7)	C4—C5	1.401 (14)
Cl1—C9	1.722 (5)	C4—H4	0.9300
S1—O4	1.413 (4)	C5—C6	1.368 (12)
S1—O3	1.420 (4)	C5—H5	0.9300
S1—N1	1.626 (5)	C6—H6	0.9300
S1—C1	1.767 (6)	C7—C8	1.371 (7)
O2—C13	1.206 (6)	C7—C12	1.386 (7)
O1—C13	1.315 (6)	C8—C9	1.383 (7)
O1—H1O	0.8200	C8—H8	0.9300
N1—C7	1.407 (6)	C9—C10	1.396 (7)
N1—H1	0.8600	C10—C11	1.388 (7)
C1—C6	1.380 (9)	C10—C13	1.489 (7)
C1—C2	1.384 (9)	C11—C12	1.360 (7)
C2—C3	1.371 (9)	C11—H11	0.9300
C3—C4	1.326 (14)	C12—H12	0.9300
C3—H3	0.9300		
			
O4—S1—O3	119.6 (3)	C5—C6—C1	121.5 (8)
O4—S1—N1	108.3 (3)	C5—C6—H6	119.3
O3—S1—N1	104.7 (3)	C1—C6—H6	119.3
O4—S1—C1	107.3 (3)	C8—C7—C12	119.0 (5)
O3—S1—C1	109.8 (3)	C8—C7—N1	122.9 (5)
N1—S1—C1	106.4 (2)	C12—C7—N1	118.1 (5)
C13—O1—H1O	109.5	C7—C8—C9	120.5 (5)
C7—N1—S1	125.6 (4)	C7—C8—H8	119.7
C7—N1—H1	117.2	C9—C8—H8	119.7
S1—N1—H1	117.2	C8—C9—C10	121.3 (5)
C6—C1—C2	118.7 (6)	C8—C9—Cl1	116.1 (4)
C6—C1—S1	117.2 (6)	C10—C9—Cl1	122.5 (4)
C2—C1—S1	124.1 (5)	C11—C10—C9	116.2 (5)
C3—C2—C1	120.3 (7)	C11—C10—C13	119.4 (4)
C3—C2—I1	115.4 (6)	C9—C10—C13	124.4 (5)
C1—C2—I1	124.3 (5)	C12—C11—C10	122.8 (5)
C4—C3—C2	119.8 (9)	C12—C11—H11	118.6
C4—C3—H3	120.1	C10—C11—H11	118.6
C2—C3—H3	120.1	C11—C12—C7	120.0 (5)
C3—C4—C5	122.4 (9)	C11—C12—H12	120.0
C3—C4—H4	118.8	C7—C12—H12	120.0
C5—C4—H4	118.8	O2—C13—O1	122.6 (5)
C6—C5—C4	117.2 (9)	O2—C13—C10	126.4 (5)
C6—C5—H5	121.4	O1—C13—C10	111.0 (5)
C4—C5—H5	121.4		
			
O4—S1—N1—C7	−58.9 (5)	S1—N1—C7—C8	30.4 (7)
O3—S1—N1—C7	172.4 (4)	S1—N1—C7—C12	−150.9 (4)
C1—S1—N1—C7	56.2 (5)	C12—C7—C8—C9	−1.7 (8)
O4—S1—C1—C6	−8.9 (5)	N1—C7—C8—C9	177.0 (5)
O3—S1—C1—C6	122.5 (5)	C7—C8—C9—C10	−2.8 (8)
N1—S1—C1—C6	−124.7 (5)	C7—C8—C9—Cl1	178.5 (4)
O4—S1—C1—C2	173.7 (5)	C8—C9—C10—C11	5.3 (7)
O3—S1—C1—C2	−54.9 (5)	Cl1—C9—C10—C11	−176.1 (4)
N1—S1—C1—C2	57.9 (5)	C8—C9—C10—C13	−173.7 (5)
C6—C1—C2—C3	0.5 (9)	Cl1—C9—C10—C13	4.9 (7)
S1—C1—C2—C3	177.9 (5)	C9—C10—C11—C12	−3.6 (8)
C6—C1—C2—I1	179.7 (4)	C13—C10—C11—C12	175.5 (5)
S1—C1—C2—I1	−3.0 (7)	C10—C11—C12—C7	−0.7 (8)
C1—C2—C3—C4	−0.6 (11)	C8—C7—C12—C11	3.4 (8)
I1—C2—C3—C4	−179.8 (6)	N1—C7—C12—C11	−175.3 (5)
C2—C3—C4—C5	0.9 (14)	C11—C10—C13—O2	−178.6 (5)
C3—C4—C5—C6	−1.2 (14)	C9—C10—C13—O2	0.3 (9)
C4—C5—C6—C1	1.1 (12)	C11—C10—C13—O1	0.3 (7)
C2—C1—C6—C5	−0.9 (10)	C9—C10—C13—O1	179.2 (5)
S1—C1—C6—C5	−178.4 (6)		

### Hydrogen-bond geometry (Å, °)

**Table:**

*D*—H···*A*	*D*—H	H···*A*	*D*···*A*	*D*—H···*A*
N1—H1···O2^i^	0.86	2.38	3.214 (6)	162
O1—H1O···O2^ii^	0.82	2.09	2.771 (6)	140

Symmetry codes: (i) *x*−1/2, −*y*+1/2, *z*−1/2; (ii) −*x*+3/2, *y*+1/2, −*z*+1/2.
